# Holistic Metabolomic Laboratory-Developed Test (LDT): Development and Use for the Diagnosis of Early-Stage Parkinson’s Disease

**DOI:** 10.3390/metabo11010014

**Published:** 2020-12-29

**Authors:** Petr G. Lokhov, Dmitry L. Maslov, Steven Lichtenberg, Oxana P. Trifonova, Elena E. Balashova

**Affiliations:** 1 Institute of Biomedical Chemistry, 10 Building 8, Pogodinskaya Street, 119121 Moscow, Russia; dlmaslov@mail.ru (D.L.M.); oxana.trifonova@gmail.com (O.P.T.); balashlen@mail.ru (E.E.B.); 2 Metabometrics, Inc, 651 N Broad St, Suite 205 #1370, Middletown, DE 19709, USA; sl@biobohemia.com

**Keywords:** diagnostics, laboratory-developed test, Parkinson’s disease, metabolomics, mass spectrometry, blood plasma, metabolite identification, biologic context, putatively annotated metabolites, metabolite sets, overrepresentation analysis

## Abstract

A laboratory-developed test (LDT) is a type of in vitro diagnostic test that is developed and used within a single laboratory. The holistic metabolomic LDT integrating the currently available data on human metabolic pathways, changes in the concentrations of low-molecular-weight compounds in the human blood during diseases and other conditions, and their prevalent location in the body was developed. That is, the LDT uses all of the accumulated metabolic data relevant for disease diagnosis and high-resolution mass spectrometry with data processing by in-house software. In this study, the LDT was applied to diagnose early-stage Parkinson’s disease (PD), which currently lacks available laboratory tests. The use of the LDT for blood plasma samples confirmed its ability for such diagnostics with 73% accuracy. The diagnosis was based on relevant data, such as the detection of overrepresented metabolite sets associated with PD and other neurodegenerative diseases. Additionally, the ability of the LDT to detect normal composition of low-molecular-weight compounds in blood was demonstrated, thus providing a definition of healthy at the molecular level. This LDT approach as a screening tool can be used for the further widespread testing for other diseases, since ‘omics’ tests, to which the metabolomic LDT belongs, cover a variety of them.

## 1. Introduction

Metabolomics studies have demonstrated the possibility of using the identification of metabolites for the successful diagnosis of many diseases [1]. These data give hope for the successful application of metabolomics methods in medicine. Therefore, researchers are trying to create such omics tests for disease diagnosis, risk assessment of their development, and determination of the patient’s response to treatment [2]. However, omics tests use in clinical practice is very challenging due to the complexity of most omics technologies, thus making their standardization for acceptance in clinics extremely difficult [3]. Consequently, considering omics tests as in-house laboratory-developed tests (LDTs) is the most evident solution to this problem. An LDT is a type of in vitro diagnostic test that is developed and used within the same laboratory [4] and is used to measure a wide range of substances, including nucleic acids, proteins, and low-molecular-weight compounds in different biological samples. To date, numerous LDTs have been developed for the diagnosis of various diseases, including cancers, infections, genetic disorders, and other pathologies [5,6,7,8,9,10,11]. In this work, an LDT comprising the latest advancements in metabolomics was developed. 

Recently, we established an LDT that can reveal overrepresentation of pathways, providing a basis for diagnosis [12]. The LDT workflow included blood sample preparation, high-resolution mass spectrometry, pre-processing of mass spectra, a compound annotation algorithm, and a statistical model testing the retrieved metabolic data against human pathways. The diagnostic performance of this LDT was demonstrated for the diagnosis of Parkinson’s disease (PD). Herein, the LDT was further developed by introducing information about metabolites, such as their known connection with diseases, conditions of the body, and location in the body. This improvement completes the development of the LDT, making it a holistic metabolomic test whereby the currently available data in the human metabolome database (HMDB [13]) are applied through panoramic measurement of metabolites in the biomaterial. This updated LDT uses disease-associated metabolite sets (631 diseases), pathway-associated metabolite sets (808 human metabolic pathways), metabolite sets associated with abnormal concentrations of metabolites (352 conditions), and location-based metabolite sets (110 sets based on their location in organs, tissues, and subcellular localization). Updated LDT was also tested as a diagnostic method for early-stage PD. 

PD is a widespread neurodegenerative disease. Its incidence has increased dramatically in recent years due to the aging population. Due to the etiologic and pathogenic heterogeneity of PD [14,15], the discovery of biomarkers for diagnosis of PD is complicated and has not yet been successful [16]. Metabolomics technologies that make it possible to measure the entire collection of low-molecular-weight compounds of a sample may help in this situation. Thus, it is reasonable to test the updated LDT on early-stage PD. In addition, the success of such diagnostics can more clearly confirm the validity of the metabolomic LDT.

## 2. Results

### 2.1. Mass Spectrometric Analysis of Compounds in Blood

**Figure 1:**
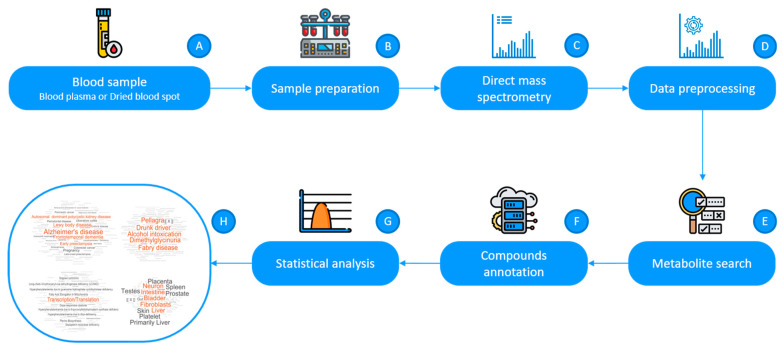
The laboratory-developed test (LDT) workflow. Blood plasma samples (or dried blood spots) are collected (**A**) and transported to the laboratory. In the laboratory, after sample preparation (**B**) and high-resolution direct mass spectrometry (**C**), the mass spectra of the blood plasma samples are obtained. The obtained masses of compounds after preprocessing (**D**) are submitted to the metabolite search block (**E**) to find metabolite identifiers from Kyoto Encyclopedia of Genes and Genomes database (KEGG) database matching the *m*/*z* values. Matched KEGG IDs is submitted to a compound annotation algorithm (**F**) [17], and the retrieved results are used for the overrepresented metabolite set analysis (**G**). Finally, overrepresented metabolite sets from an individual are visualized as a metabolite set names cloud, where the font size corresponds to the representation value (score) (**H**).

Mass spectrometric analysis, as the first analytical block of the LDT (Figure 1), generated high-resolution spectra of approximately 10,000 mass peaks of low-molecular-weight compounds in the blood plasma samples. The measured masses were submitted to the bioinformatic treatment block of the LDT that resulted in the annotation of 709 compounds (Table 1).

### 2.2. Metabolite Set Overrepresentation Patterns in the LDT Output

**Figure 2:**
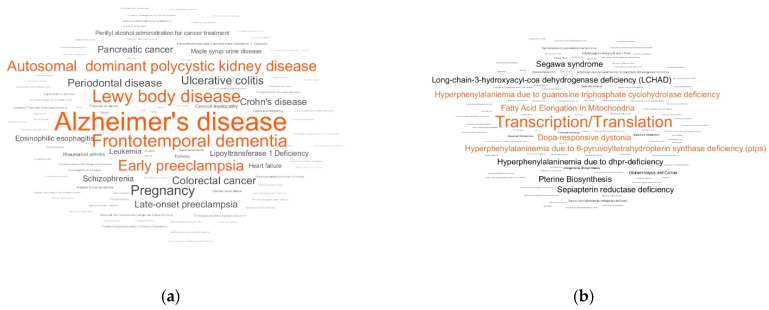

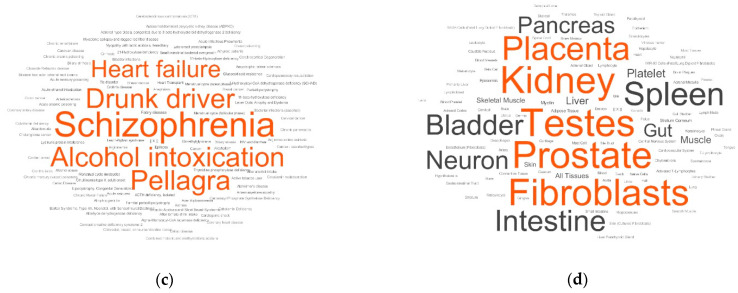
LDT output for the ‘case-control’ study of Parkinson’s disease (PD). The output is presented for disease-associated metabolite sets (**a**), pathway-associated metabolite sets (**b**), metabolite sets associated with abnormal concentrations of metabolites (**c**), and location-based metabolite sets (**d**). The top five overrepresented metabolite sets are colored in red.

A case-control comparison revealed patterns of PD in the LDT output. The LDT output was generated as a metabolite set names cloud for the controls and cases. Among the top overrepresented disease-associated metabolite sets, the diseases semantically similar to PD were presented (Alzheimer’s disease, Lewy body disease, and frontotemporal dementia; Figure 1a). The PD-relevant patterns were also found in the pathway-associated metabolite sets (Figure 2b) and the metabolite sets associated with abnormal concentrations of metabolites (Figure 2c). With the use of the location-based metabolite sets, excluding a neuron-associated set, it was difficult to associate the top overrepresented sets with PD directly (Figure 2d).

The metabolite set representation scores for the control samples, samples from PD patients, and the top 20 overrepresented metabolite sets for the PD patients are listed in Table 2.

### 2.3. Diagnostic Performance of the LDT

Using the LDT, the diagnosis of PD based on diagnostic score reached an accuracy of 73% (Table 2). It is noteworthy that the high diagnostic performance practically did not depend on the type of metabolite sets used, that is, whether the disease-associated sets, pathway-associated sets, abnormal concentration sets, or localization-based sets were used did not matter. This is explained by the fact that the same metabolites, indicating PD, were applied to these groups. 

Upon analysis of the diagnostic performance based on overrepresented metabolite sets, it was noted that a neurodegenerative disease of the central nervous system could be suspected in patients. Thus, the metabolite sets of Alzheimer’s disease, Lewy body disease, and frontotemporal dementia were overrepresented. Almost all of the overrepresented pathways (Table 2) were also relevant to PD. Thus, dysregulation of transcription and translation is described in PD [18,19]. Moreover, it is a known fact that dopamine level is connected with PD [20]. The role of lipid metabolism and mitochondria is also described for PD [21]. Furthermore, overrepresented pterin synthesis is directly connected with neurotransmitters; Segawa syndrome together with guanosine triphosphate cyclohydrolase deficiency is related to Dopa-responsive dystonia; and 6-pyruvoyltetrahydropterin synthase deficiency is a neurodegenerative disease that, similar to dihydropteridine reductase (DHPR) deficiency, is treated by levodopa. Overexpression was also observed in sets of metabolites associated with internal organs, for example, high overexpression in a set of metabolites associated with colorectal cancer. Synucleinopathy can explain such overexpression. Synucleinopathy develops in various parts of the nervous system at PD and leads to denervation of the heart, disturbances in the large intestine, esophagus, kidneys, etc. A metabolite set associated directly with PD was not presented in the top list of overrepresented disease-related sets. Perhaps this is due to the lack of such a set that is applicable to the LDT.

The overrepresented metabolite sets associated with abnormal concentrations also contributed to the diagnostic performance of the LDT. Regarding the top overexpressed sets (schizophrenia, alcohol intoxication and drunk driver, pellagra, etc.; see Table 2), it is known that dopamine and dopaminergic neurons play an important role in schizophrenia, as well as in PD [22,23]. Moreover, it has been argued that a functional excess of dopamine or oversensitivity of certain dopamine receptors is one of the causal factors in schizophrenia. In schizophrenia, the antipsychotic effects of traditional ‘neuroleptic’ drugs, such as chlorpromazine, are highly correlated with their ability to block dopamine receptors and reduce the effects of dopamine. The overrepresentation of alcohol intoxication and drunk driver sets may be explained by the fact that alcohol has a powerful effect on dopamine activity in the brain, which has been revealed in animals [24] and human studies [25]. Pellagra is due to a diet that does not contain enough niacin and tryptophan, which, in turn, can be converted into serotonin and are altered in PD [26]. 

### 2.4. Diagnosis of PD by the LDT

**Figure 3:**
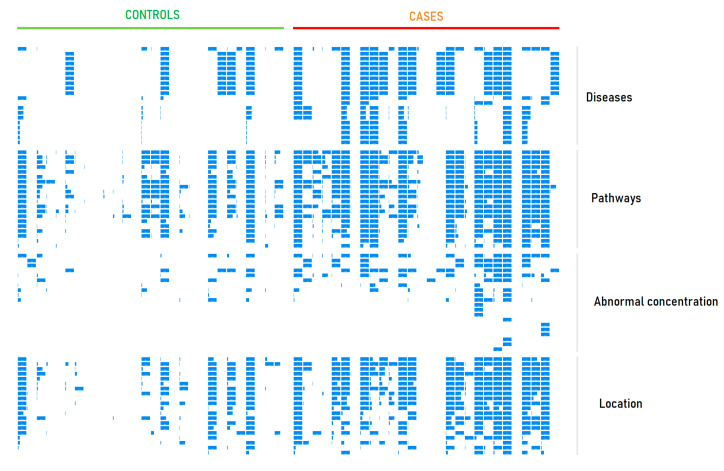
Metabolite set representation scores produced by the laboratory-developed test (LDT) for control subjects and patients with Parkinson’s disease (PD). The rows correspond to the metabolite sets presented in Table 2. The widest bands correspond to a metabolite set representation score of 75.

**Figure 4:**
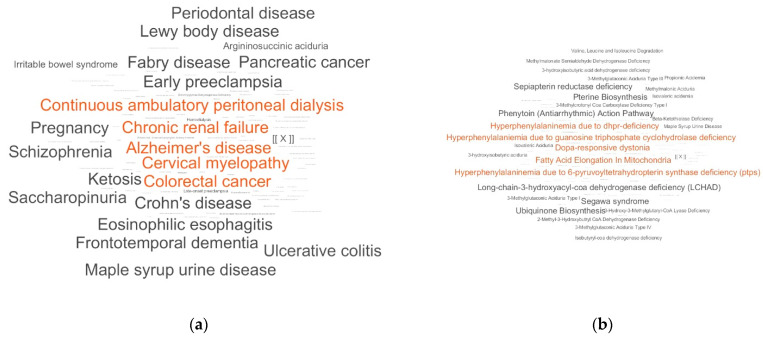

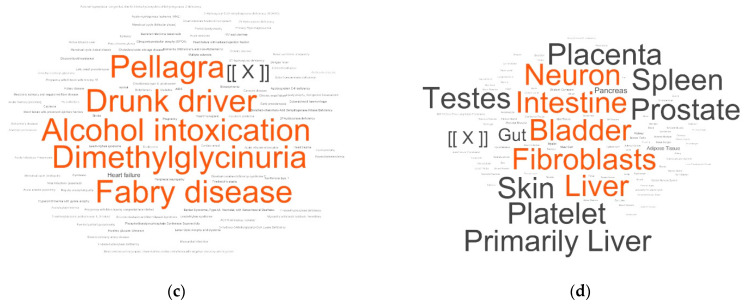
The laboratory-developed test (LDT) outputs for a person with Parkinson’s disease (PD). The LDT outputs for the overrepresentation pattern in disease-associated metabolite sets (**a**), pathway-associated metabolite sets (**b**), metabolite sets associated with abnormal concentrations (**c**), and location-based metabolite sets (**d**). [[X]] is a marker corresponding to a representation score of 50. The overrepresented metabolite sets with the top five scores are colored in red.

Figure 3 shows the metabolite set representation scores for each participant in this study. This figure shows that diagnostics based on the metabolite set representation score is possible. Unlike analysis of the case-control sets, which reveals the common group patterns, personal data analysis is more complicated. Aged patients have a whole range of diseases leading to the overrepresentation of different metabolite sets, thus making diagnosis very difficult. However, for some individuals, it is quite possible. Figure 4 presents the LDT output as a metabolite set names cloud for one person. 

The overrepresentation of metabolite sets associated with neurodegenerative diseases (Figure 4a), together with the overrepresentation of the Dopa-responsive dystonia pathway (Figure 4b), which is a movement disorder characterized by muscle tone and Parkinsonian features, the change in the concentration of substances matching alcohol intoxication (Figure 4c), and priority localization of such metabolites (Figure 4c) with an abnormal concentration in neurons, would allow health professionals to suspect neurodegenerative disease and motivate them to go deeper and to obtain additional support for a PD diagnosis. An overrepresentation of the metabolic sets associated with Fabry disease [27], pellagra, which, as mentioned above, has a connection with PD, as well as many Dopa-related pathways (Segawa syndrome, pterine biosynthesis, sepiapterin reductase deficiency, guanosine triphosphate cyclohydrolase deficiency, 6-pyruvoyltetrahydropterin synthase deficiency (PTPS), and DHPR-deficiency) was observed. In other words, the LDT indicates which direction to search, thus helping the clinician to select of confirmatory, targeted tests. For this patient, an appointment for a single-photon emission computerized tomography scan is fully justified, and PD will be diagnosed.

### 2.5. LDT Output for a ‘Healthy’ Individual

**Figure 5:**
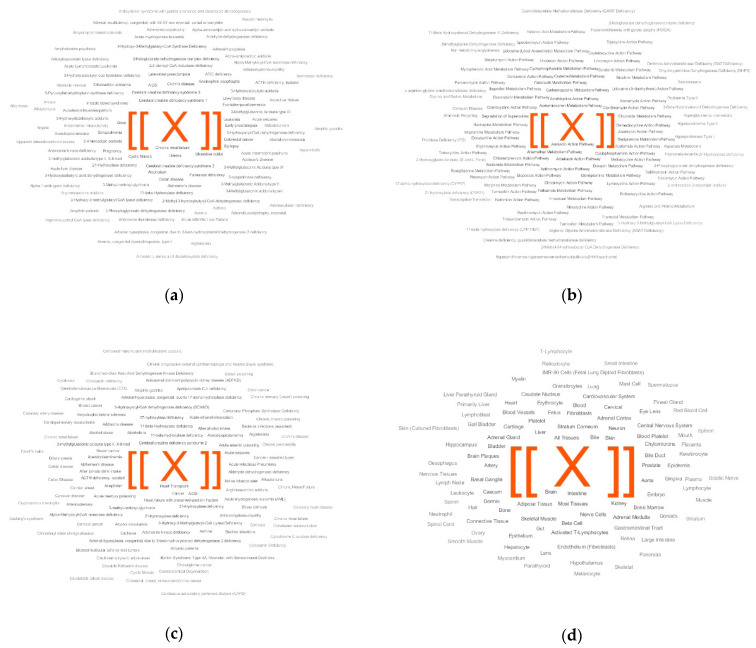
The laboratory-developed test (LDT) outputs for a ‘healthy’ individual. The LDT outputs for the overrepresentation pattern in disease-associated metabolite sets (**a**), pathway-associated metabolite sets (**b**), metabolite sets associated with abnormal concentrations (**c**), and location-based metabolite sets (**d**). No overrepresented metabolite sets were detected, thus confirming that the individual does not have any deviation in the metabolite composition of their blood. [[X]] is a marker corresponding to a representation score of 50.

In the control group, there were individuals for whom the LDT did not reveal any overrepresentation in the metabolite sets. Thus, from the point of view of metabolic processes, these people can be considered ‘healthy,’ since the composition of low-molecular-weight compounds in their blood corresponds to the age norm. An example of the LDT results for such a person is presented in Figure 5.

## 3. Discussion

The Food and Drug Administration (USA) considers LDTs as tests that are designed, manufactured, and used inside the same laboratory [4]. It simplifies the implementation of metabolomics-based tests, bringing protocols and standardization activities to single laboratory routines. The developed LDT is based on direct mass spectrometry of blood plasma, which has been widely used in metabolomics and, in particular, in the laboratory where LDTs were developed for the study of cancers [28,29,30,31,32], diabetes [33], and PD [34]. This type of mass spectrometry is characterized by a high processing speed and a relatively high reproducibility [35,36,37], which are important for the use of mass spectrometry in the clinic. The mass spectrometry data processing, like peak alignment and data standardization, was specially developed for high-resolution mass spectra and successfully used for many years in studies of blood plasma [29,38] and now is implemented in the LDT. 

Generally, mass spectrometry allows the detection of hundreds of compounds in metabolomics studies, which is crucial for obtaining biochemical information [39]. Unfortunately, the vast majority of compounds in the sample remain unknown [40]; current annotation methods require a clear mass spectrometric picture of compounds or its fragments, which can be obtained only for well-separated and abundant metabolites. In the LDT described in this study, a recently developed biochemical context-driven annotation is realized for annotation of compounds, which uses the knowledge of their biotransformation in metabolic pathways. This approach was introduced by Rogers and coworkers [41] and further updated by Silva and coworkers [42]. Later, the suitability of this approach for blood plasma samples was also demonstrated [17]. This updated algorithm was implemented in the LDT, which allowed the annotation of more than 700 metabolites per sample. The obtained metabolite annotations were classified as putatively annotated compounds (level 2 of metabolite identification), according to the Metabolomics Standards Initiative standard [43], because two independent orthogonal features of each metabolite were used for annotation (accurate mass tag and biochemical context). Thus, annotation results do not include the most robust identifications at level 1, which is acceptable for medical purposes and requires a chemical standard for identification. Obviously, for big data, to which the metabolomics data relates, a level 1 often is impossible, thus making it reasonable to use the described approach as a screening technology, which helps the clinician to optimize the selection of confirmatory, secondary tests [44].

The selection of PD to test this LDT was not an accident. Previously, it has been shown that an LDT can reveal the pathway overrepresentation efficient for the diagnosis of PD [12], thus making it reasonable to complete LDT development by introducing metabolic data about diseases, different organism conditions, and metabolite location. Figure 3 shows the representation scores for each participant in this study and each metabolite set. This figure confirms that each metabolite set contributes to diagnostics, and the accuracy of such kind diagnostics is 73% (Table 3). It should be noted, the early laboratory diagnosis of PD is currently unavailable and urgently needed for effective therapy [45,46,47]. However, the multifactorial nature of PD complicates the development of conventional biomarker-based tests. The clinical application of ‘panoramic’ methods, to which metabolomic methods are related, have the complex workflow that makes their standardization and following registration illusive. The usage of the LDT in such a situation overcomes this obstacle because all of the LDT-related routines are located in a single laboratory. 

The developed LDT uses currently available data on the concentrations of metabolites in humans and uses them to analyze panoramically measured blood composition data. Along with the use of modern data processing algorithms, it can be argued that the LDT is an omics test that demonstrates the current diagnostic capabilities of metabolomics, the most obvious of which are as follows:

- **Confirmation of a person’s healthy state**. This option of the LDT is the most obvious; the output of the LDT in this case is self-explanatory and comprehensively confirms human health at the molecular level. The LDT shows that the detected deviations in the blood composition do not form any patterns specific to a disease or pathology. So, the LDT is ready for use to determine wellness and longevity. It is expected that the healthy state can be confirmed by the LDT and that any abnormalities that will appear at the molecular level can be detected in a timely manner, which lays the foundation for a long and quality life.
- **Score-based diagnostics.** Score-based diagnostics requires control samples and samples from a cohort of patients with disease. The advantage of such diagnostics is the absence of human error in diagnosis and possible full automation.
- **Disease diagnosis based on metabolite set overrepresentation (i.e., without diagnostic scoring).** This option of the LDT is ready to use (i.e., cohorts are not required) for the diagnosis of a wide diversity of diseases. The metabolite set names cloud allows visualization of the LDT output data that a physician can interpret. An example of this is demonstrated in this paper for the diagnosis of PD, although, among the LDT outputs, there were also results that were difficult to interpret. It is possible that the effectiveness of the LDT output interpretation will increase as further LDT output data are accumulated. Most importantly, the LDT is panoramic in terms of measuring substances and untargeted in terms of diagnosing diseases, which in the end makes it especially valuable.

The main disadvantage of the LDT is its complexity, which leads to the lack of strictly standardized protocols and direct dependence on the equipment used and the experience of the staff working on it. In general, therefore, the LDT is implemented in a single laboratory and, as a rule, is not translated to other laboratories. But this problem can be substantially ignored by the compatibility of the mass spectrometric measurement in the LDT with a dried blood spot [48] because a dried blood spot can be obtained without assistance at home and transported on a blood sample card at room temperature by mail, thus making the LDT available almost everywhere. It seems that such a type of laboratory diagnostic method that does not require direct contact with people during blood sampling will be relevant in light of the pandemic that has swept the world.

## 4. Materials and Methods

### 4.1. Mass Spectra of Blood Plasma

Samples of blood plasma used in this study were taken from the previously published study [34]. Table 3 presents the clinical characteristics of the cohort.

**Table 3:** Study cohort characteristics.

Characteristics	Values	
	Subjects with PD	Control Subjects
Number	28	28
Gender (male/female)	14/14	14/14
Age (years; mean ± s.d. (range))	62.6 ± 8.6 (37–77)	62.8 ± 8.7 (45–77)
PD stages (1/1.5/2/2.5) ^1^	6/6/12/4	-

^1^ PD stages are according to Hoehn and Yahr scale [49].

Samples were analyzed with a maXis hybrid quadrupole time-of-flight mass spectrometer with an electrospray ionization source as described in the previously published study [12]. Normalization of mass peak intensities was performed as described previously [38]. The alignment of the *m*/*z* values of the mass peaks to the different mass spectra was performed as described previously [29]. The resulting *m*/*z* values with a nonzero mass peak intensity for more than nine samples (removes noise and suspect data) were submitted to the metabolite search block of the in-house software. 

### 4.2. Compound Annotation

The search for correspondence of each mass peak to metabolite identifiers was done by the metabolite search block of the in-house software as described in the previous publication [12]. The HMDB (www.hmdb.ca) was used as the source of the *m*/*z* values and identifiers from the Kyoto Encyclopedia of Genes and Genomes database (KEGG IDs). A compound annotation algorithm was recently developed and described in detail [42]. This algorithm uses metabolic pathway data and allows for the effective annotation of low-molecular-weight blood components (metabolome) with relatively high speed. In the list of compound names, many candidates, on average, were associated with one mass. The main task of the algorithm is to compare the obtained experimental data, i.e., mass spectra, with the available information on biochemical pathways and to decline all false candidates. It is known that the concentrations of compounds involved in the same pathways correlate [50]. Thus, if mass spectrometry data for a set of samples are available, the correlation between the mass of interest and other mass peaks can be found. The masses of these correlating peaks can also be associated with a set of compounds in which their locations in the metabolic pathway must be bunched around the compound with the true annotation. The details of the application of this algorithm for blood plasma samples have been described previously [17]. The next release of this algorithm with updated source code to make it more suitable for blood samples was recently used in the LDT [12] and applied in this work.

### 4.3. Metabolite Set Overrepresentation Analysis

Analyzed HMDB data were used to compile metabolite sets: disease-associated metabolite sets (631 diseases), pathway-associated metabolite sets (808 human metabolic pathways), metabolite sets associated with the abnormal concentrations of metabolites (352 conditions), and location-based metabolite sets (110 sets based on location in organs, tissues, and subcellular localization). Selected metabolites in the case-control or individual sample studies were projected on these metabolite sets, and the obtained results were compared with the projections, which were performed 30,000 times with the same number of randomly selected metabolites. The obtained results were normalized to produce a metabolite set representation score for each set as described previously [12]. This score also was used to visualize the results as a metabolite set names cloud in which the font size is related. The scores for the top 20 overrepresented metabolite sets were summarized to produce the final diagnostic score for each person who participated in the study. 

To reveal the PD-associated pattern in metabolite sets, the mean value for representation scores for cases were compared with those of the controls. 

To estimate diagnostic performance of LDT, as well as to estimate the metabolic data projection on the separate metabolite sets, the *perfcurve* function of the MATLAB program was used. This function presents the accuracy, sensitivity, and specificity for each point of receiver operating characteristic (ROC) curve and selects the optimal values.

### 4.4. Analysis of Individual Samples by the LDT

To reveal mass peaks belonged to metabolites with abnormal concentration, the Z-score calculation and the leave-one-out approach were applied for mass peak intensities as described previously [51]. This method involves the one-by-one removal of each data point (sample) from the dataset and recalculation of the model parameters based on the remaining data. The model is then tested by the excluded sample. 

The LDT workflow used to analyze blood plasma samples is presented in Figure 1. The in-house software, as a part of the LDT, was implemented in MATLAB and was used for data pre-processing, database searching, and overrepresentation analysis. To perform all calculations, a Lenovo (Intel^®^ Xeon^®^ E-2176M CPU 2.70 GHz, Windows 10 Pro) computer was used.

## 5. Conclusions

LDTs or, more familiarly, ‘home brew’ tests, have been around for decades. Traditionally, their scope is small for low-risk diagnostic applications. Today, more complex LDTs can be used. Being a ‘direct-to-customer’ test, LDTs provide clinical results to a wide range of customers: physicians and their patients, researchers, citizen scientists, and simply educated people. While the current work demonstrated a metabolomic LDT for supporting the diagnosis of PD, the omics nature of the LDT suggests that it can be used for a variety of diseases. Information, in the name cloud form, in the LDT output about the state of the organism can be easy and quick to read by a wide range of customers although such an output is formed by metabolic big data. Diagnostics through the use of the metabolomic LDT for a wide variety of diseases on a single dried blood spot obtained at home lays the groundwork for improvements in terms of accessibility, price, and versatility of laboratory diagnostics, which can lead to an improvement in people’s living standards. 

## Figures and Tables

**Table 1:** Variables associated with this study.

Parameter	Value
Detection mass range (*m*/*z*)	45–900
Detected compound mass peaks (mean ± s.d.)	9664 ± 620 ^1^
Masses submitted to metabolite search block	14,857
‘Mass peak/metabolite name’ pairs submitted to the annotation algorithm	31,724
Mass peaks with annotated compound(s)	2741
Unique compound annotations	709

^1^ average ± standard deviation.

**Table 2:** Criteria for laboratory-developed test diagnostics for Parkinson’s disease.

Metabolite Set	Representation Score		Over-Representation	Diagnostic Performance		
	Controls	Cases		Sensitivity	Specificity	Accuracy
**Disease-associated metabolite sets**						
Alzheimer’s disease	21.9	53.8	31.9	75.0	71.4	73.2
Frontotemporal dementia	24.4	53.0	28.6	75.0	67.9	71.4
Lewy body disease	24.4	53.0	28.6	75.0	67.9	71.4
Early preeclampsia	15.0	42.5	27.5	67.9	71.4	69.6
Autosomal dominant polycystic kidney disease	12.6	40.1	27.4	60.7	78.6	69.6
Pregnancy	16.8	43.6	26.8	75.0	67.9	71.4
Ulcerative colitis	27.5	53.0	25.5	78.6	60.7	69.6
Colorectal cancer	26.8	52.0	25.1	89.3	57.1	73.2
Periodontal disease	20.8	45.8	25.0	60.7	78.6	69.6
Pancreatic cancer	23.2	47.7	24.5	64.3	71.4	67.9
Late-onset preeclampsia	12.1	36.7	24.5	60.7	75.0	67.9
Crohn’s disease	25.9	50.0	24.2	71.4	64.3	67.9
Schizophrenia	25.0	48.3	23.2	64.3	71.4	67.9
Eosinophilic esophagitis	28.5	51.1	22.6	64.3	71.4	67.9
Lipoyltransferase 1 deficiency	10.2	32.7	22.5	71.4	67.9	69.6
Leukemia	9.0	31.3	22.3	75.0	75.0	75.0
Maple syrup urine disease	16.3	37.5	21.1	67.9	67.9	67.9
Perillyl alcohol administration for cancer treatment	12.9	33.9	21.1	67.9	71.4	69.6
Heart failure	3.3	24.0	20.7	53.6	85.7	69.6
Rheumatoid arthritis	1.6	20.8	19.2	46.4	89.3	67.9
Parameters for the whole group of metabolite sets:				78.6	60.7	69.6
**Pathway-associated metabolite sets**						
Transcription/translation	17.4	43.2	25.7	71.4	71.4	71.4
Dopa-responsive dystonia	13.4	34.8	21.4	46.4	82.1	64.3
Fatty acid elongation in mitochondria	13.4	34.8	21.4	46.4	82.1	64.3
Hyperphenylalaniemia due to guanosine triphosphate cyclohydrolase deficiency	13.4	34.8	21.4	46.4	82.1	64.3
Hyperphenylalaninemia due to 6-pyruvoyltetrahydropterin synthase deficiency (PTPS)	13.4	34.8	21.4	46.4	82.1	64.3
Hyperphenylalaninemia due to DHPR-deficiency	13.4	34.8	21.4	46.4	82.1	64.3
Long-chain-3-hydroxyacyl-coa dehydrogenase deficiency (LCHAD)	13.4	34.8	21.4	46.4	82.1	64.3
Pterine biosynthesis	13.4	34.8	21.4	46.4	82.1	64.3
Segawa syndrome	13.4	34.8	21.4	46.4	82.1	64.3
Sepiapterin reductase deficiency	13.4	34.8	21.4	46.4	82.1	64.3
Glutaminolysis and cancer	4.1	22.0	17.8	57.1	75.0	66.1
Ubiquinone biosynthesis	0.1	16.1	16.0	28.6	92.9	60.7
Aspartate metabolism	4.2	19.4	15.2	57.1	78.6	67.9
Canavan disease	4.2	19.4	15.2	57.1	78.6	67.9
Hypoacetylaspartia	4.2	19.4	15.2	57.1	78.6	67.9
2-Hydroxyglutric aciduria (D and L Form)	1.0	16.1	15.1	25.0	100.0	62.5
4-Hydroxybutyric Aciduria/succinic semialdehyde Dehydrogenase deficiency	1.0	16.1	15.1	25.0	100.0	62.5
Glutamate metabolism	1.0	16.1	15.1	25.0	100.0	62.5
Homocarnosinosis	1.0	16.1	15.1	25.0	100.0	62.5
Hyperinsulinism-hyperammonemia syndrome	1.0	16.1	15.1	25.0	100.0	62.5
Parameters for the whole group of metabolite sets:				82.1	64.3	73.2
**Abnormal concentration-associated metabolite sets**						
Schizophrenia	10.9	33.6	22.7	64.3	75.0	69.6
Alcohol intoxication	2.7	24.1	21.4	32.1	96.4	64.3
Drunk driver	2.7	24.1	21.4	32.1	96.4	64.3
Pellagra	13.4	34.8	21.4	46.4	82.1	64.3
Heart failure	3.3	24.0	20.6	53.6	89.3	71.4
Fabry disease	8.0	18.8	10.7	25.0	89.3	57.1
Epilepsy	0.5	6.8	6.3	21.4	92.9	57.1
Heart transplant	5.0	11.0	6.0	50.0	67.9	58.9
Lesch-Nyhan syndrome	0.7	5.8	5.1	75.0	57.1	66.1
Dimethylglycinuria	4.7	9.8	5.1	32.1	85.7	58.9
Menstrual cycle (follicular phase)	0.0	2.7	2.7	42.9	82.1	62.5
Menstrual cycle (luteal phase)	0.0	2.7	2.7	42.9	82.1	62.5
Menstrual cycle (midcycle)	0.0	2.7	2.7	42.9	82.1	62.5
ACTH deficiency, isolated	0.0	2.7	2.7	39.3	85.7	62.5
Small intestinal bacterial overgrowth	0.0	2.7	2.7	28.6	89.3	58.9
Crohn’s disease	0.0	2.7	2.7	28.6	89.3	58.9
HIV and diarrhea	0.0	2.7	2.7	28.6	89.3	58.9
Glucocorticoid resistance	0.0	2.7	2.7	35.7	89.3	62.5
Tic disorder	0.0	2.7	2.7	35.7	89.3	62.5
Thymidine phosphorylase deficiency	0.0	2.7	2.7	39.3	82.1	60.7
Parameters for the whole group of metabolite sets:				82.1	64.3	73.2
**Location-based metabolite sets**						
Testes	11.0	39.4	28.4	57.1	85.7	71.4
Prostate	22.8	51.2	28.4	67.9	75.0	71.4
Kidney	15.8	44.1	28.3	67.9	75.0	71.4
Fibroblasts	19.5	47.8	28.2	78.6	67.9	73.2
Placenta	14.6	42.1	27.5	64.3	78.6	71.4
Spleen	12.8	40.1	27.3	60.7	82.1	71.4
Intestine	19.7	46.4	26.7	67.9	71.4	69.6
Bladder	16.4	42.7	26.4	71.4	75.0	73.2
Neuron	11.1	36.8	25.6	78.6	71.4	75.0
Pancreas	15.5	40.6	25.1	71.4	71.4	71.4
Gut	8.2	31.6	23.4	60.7	78.6	69.6
Platelet	7.0	27.6	20.5	60.7	75.0	67.9
Liver	15.9	35.7	19.8	64.3	71.4	67.9
Muscle	13.4	32.8	19.4	71.4	78.6	75.0
Skeletal muscle	10.8	28.0	17.2	60.7	75.0	67.9
All tissues	13.6	30.4	16.8	75.0	60.7	67.9
Skin	3.5	20.1	16.6	64.3	64.3	64.3
Myelin	5.3	18.4	13.1	60.7	85.7	73.2
Adipose tissue	5.9	17.7	11.8	64.3	71.4	67.9
Stratum corneum	1.1	12.5	11.4	50.0	89.3	69.6
Parameters for the whole group of metabolite sets:				71.4	71.4	71.4
Parameters for the whole groups of metabolite sets:				89.3	57.1	73.2

## Data Availability

The data presented in this study are openly available in FigShare at doi 10.6084/m9.figshare.13621277.
